# Periodontal status and microbial profiles across the clinical spectrum of liver cirrhosis

**DOI:** 10.1080/20002297.2026.2678639

**Published:** 2026-05-26

**Authors:** J. B. O. Batista, E. H. de Souza Oliveira, L. A. M. Penteado, V. X. Nascimento, S. Matsumura, J. M. M. Silva, D. F. Nóbrega, B. Retamal-Valdes, M. Feres, S. M. S. Ferreira

**Affiliations:** a Hospital Universitário Professor Alberto Antunes (HUPAA), Maceió, AL, Brazil; b Centro Universitário CESMAC, Maceió, AL, Brazil; c Department of Oral Medicine, Infection, and Immunity, Harvard School of Dental Medicine, Boston, MA, USA; d Department of Periodontology, Graduate School of Medical and Dental Sciences, Institute of Science Tokyo, Tokyo, Japan; e Department of Periodontology, RS Master Saúde, Aruja, SP, Brazil; f Department of Periodontology, Faculty of Dentistry, Universitas Gadjah Mada, Yogyakarta, Indonesia; g Department of Dental Sciences, Technical University of Oruro, Oruro, Bolivia; h Dental Research Division, Department of Periodontology, Universidade Guarulhos (Guarulhos University), Guarulhos, SP, Brazil

**Keywords:** Liver cirrhosis, periodontal diseases, periodontitis, subgingival plaque, microbiota, hybridization, nucleic acid

## Abstract

**Objectives:**

Periodontitis is common in patients with cirrhosis, but this association remains underexplored. This cross-sectional study aimed to assess periodontal conditions and characterize periodontal biofilm composition in individuals with liver cirrhosis.

**Methods:**

Adults with liver cirrhosis were enrolled. Periodontal parameters were recorded, and supragingival and subgingival biofilm samples were collected. Forty bacterial species were quantified using checkerboard DNA–DNA hybridization. Findings were stratified by Child–Pugh class and MELD score.

**Results:**

Forty-five patients were included; 88.9% exhibited periodontal disease (57.8% gingivitis on a reduced periodontium, 31.1% periodontitis), and 11.1% were periodontally healthy. Periodontal diagnosis was not associated with liver-disease severity (*p* > 0.05). Gingivitis showed the highest supragingival bacterial load, with enrichment of orange- and red-complex pathogens, particularly *Tannerella forsythia*. Patients with compensated liver disease (Child–Pugh A; MELD < 15) showed higher counts of *Aggregatibacter actinomycetemcomitans* and *Fusobacterium nucleatum* ssp. *nucleatum* (*p* < 0.05). Among patients with periodontitis, higher subgingival proportions of red-complex pathogens were observed in those with MELD < 15.

**Conclusions:**

Periodontal disease was highly prevalent in patients with cirrhosis. Although periodontal diagnosis was not associated with liver-disease severity, distinct microbiological patterns were observed. These findings highlight the potential relevance of periodontal monitoring in patients with cirrhosis and warrant further investigation in larger, longitudinal studies.

## Key messages

- In patients with liver cirrhosis, gingivitis was characterized by the highest supragingival bacterial burden and marked enrichment of orange- and red-complex species, particularly *Tannerella forsythia*, suggesting this clinical stage represents a state of heightened supragingival dysbiosis.
- Periodontitis patients with compensated liver disease (MELD < 15) harboured higher subgingival proportions of red-complex pathogens than those with more advanced cirrhosis, indicating a non-linear, host-modulated relationship between hepatic dysfunction and periodontal microbial ecology.
- Distinct microbial signatures were observed across supragingival and subgingival niches and liver disease severity strata, supporting the value of niche-specific microbiological profiling in medically compromised populations.

## Introduction

Cirrhosis, the end stage of chronic liver disease, is a major global health problem and one of the leading causes of morbidity and mortality worldwide [1,2]. It results from persistent hepatocellular injury that activates hepatic stellate cells and drives excessive collagen and extracellular matrix deposition, ultimately leading to progressive fibrosis and nodular regeneration throughout the liver [3–5]. Beyond hepatic dysfunction, cirrhosis has profound systemic consequences, impairing immune competence, microbial clearance, and overall quality of life [6,7]. Importantly, these systemic disturbances extend to the oral cavity, although this aspect of the disease remains underexplored [8].

Oral health is increasingly recognized as a relevant component of systemic well-being, and cirrhotic patients often display compromised oral conditions. Previous studies have shown that individuals with cirrhosis report poorer oral hygiene habits and worse self-perceived oral health than the general population [8,9]. Consistently, poor oral hygiene practices in this group are associated with an increased prevalence and incidence of gingivitis and periodontitis [10,11]. Gingivitis, an inflammatory disease restricted to the gingival tissues, may progress in susceptible hosts to periodontitis, an inflammatory and destructive condition affecting the tooth-supporting structures that, without proper treatment, may lead to tooth loss [12].

Periodontitis is driven by dysbiosis of the periodontal microbiome, which reflects an imbalance between the microbial community and the host immune-inflammatory response [13]. Numerous systemic and behavioural factors, including impaired immune function, stress, low educational level, limited access to dental care, salivary dysfunction, alcohol consumption, diabetes mellitus and smoking, can modulate this interaction and exacerbate disease susceptibility [14]. In cirrhotic patients, many of these risk factors converge, increasing the likelihood of oral dysbiosis and periodontal breakdown. In fact, epidemiologic studies have reported a higher incidence of periodontitis in cirrhotic patients, ranging from 25% to 68% [15].

Despite this biological plausibility, the interplay between cirrhosis [14], dysbiosis, and periodontal disease has been insufficiently investigated [16,17]. Moreover, the contribution of oral diseases, including periodontal diseases, to the clinical course of cirrhosis remains poorly understood [18], despite suggestions that hepatic dysfunction may exacerbate oral disease through impaired clearance of bacteria, endotoxins, and inflammatory mediators [19]. Only one study to date has examined the subgingival periodontal microbiota in 21 cirrhotic patients with periodontitis [20].

While the available evidence and biological plausibility suggest potential links between cirrhosis, oral dysbiosis, and periodontal disease, the current literature is largely observational, and the relationships described should be interpreted as associative rather than causal. Notably, this association has not yet been investigated within distinct periodontal microbial niches, such as supragingival and subgingival biofilms, or across different stages of liver-disease severity.

Given the scarcity of studies addressing the periodontal microbiota in cirrhotic patients [20], this study aimed to explore, in a cross-sectional design, periodontal conditions and the supragingival and subgingival microbiota, focusing on associations between clinical and microbial patterns across periodontal diagnoses and stages of liver disease severity.

## Materials and methods

### Study design

This observational, analytical, cross-sectional study was conducted at the hepatology clinic of Hospital Universitario Professor Alberto Antunes (Maceio, Alagoas, Brazil). The study protocol was approved by the Centro Universitario CESMAC Research Ethics Committee (Opinion No. 3,077,999). All participants were informed about the study procedures and provided written informed consent.

The inclusion criteria included: adults (≥18 years old) with a clinical diagnosis of alcoholic or non-alcoholic liver cirrhosis were eligible. Per institutional routine, cirrhosis was established by physician assessment integrating medical history, physical examination, laboratory tests, ultrasonography, and/or liver biopsy (as applicable).

The exclusion criteria were: fewer than six teeth [21]; any condition precluding periodontal examination (e.g. trismus with severe limitation of mouth opening); cognitive impairment; use of immunosuppressants or receipt of periodontal treatment within the prior 6 months; antibiotic use within the prior 3 months; any malignant disease; systemic conditions known to influence periodontal status (e.g. diabetes mellitus, acquired immunodeficiency syndrome); and acute critical illness (e.g. overt hepatic encephalopathy or active bleeding from ruptured esophageal varices). Cognitive impairment was screened using the Mini-Mental State Examination [22–24].

### Sample size calculation

Given the exploratory, hypothesis-generating nature of this cross-sectional study and the absence of reliable prior estimates for variability and effect sizes across the targeted bacterial panel in cirrhotic patients, no *a priori* sample size calculation was performed. Therefore, a convenience sample of consecutively eligible patients during the recruitment period was included.

### Sociodemographic and liver-related data collection

Two trained researchers extracted data from medical records and participant interviews onto standardized case-report forms, including: sociodemographic variables; cirrhosis etiology; disease severity [Child–Pugh class and Model for End-Stage Liver Disease (MELD) score]; cirrhosis complications; comorbidities; current medications; alcohol consumption; and relevant laboratory tests.

The Child–Pugh system comprised two clinical (ascites, encephalopathy) and three laboratory criteria [total bilirubin (mg/dL), albumin (g/dL), INR) to classify patients as A, B or C; Class A (5–6) is well-compensated, Class B (7–9) has significant functional compromise, and Class C (10–15) is decompensated [25]. MELD is a numerical score derived from total bilirubin, INR and serum creatinine [3] and is used for liver transplant allocation; for instance, MELD ≥15 is associated with an elevated 3-month mortality risk.

### Assessment of oral-health behaviours and self-perception

A trained researcher administered a validated oral-health behaviour and self-perception questionnaire [26]. The items covered behaviours (oral hygiene practices, sugar intake, dental attendance pattern, type of dental service, reason for last visit and smoking status) and self-perception (perceived need for dental treatment, toothache in the past 6 months, satisfaction with the mouth and teeth).

### Periodontal clinical assessment

Periodontal status was determined by a comprehensive clinical examination performed by a trained dentist using a UNC-15 probe (Hu-Friedy®, Chicago, IL, USA). Examiner calibration entailed two full-mouth examinations separated by a 40-min interval. Calibration success was predefined as an intraclass correlation coefficient (ICC) ≥0.75. ICCs were computed in BioEstat 5.0; the examiner achieved excellent reproducibility (ICC = 0.88; *p* < 0.0001).

Six sites per tooth (distobuccal, mid-buccal, mesiobuccal, distolingual/palatal, mid-lingual/palatal and mesiolingual/palatal), excluding third molars, were assessed for: gingival margin position (GMP), probing depth (PD), clinical attachment level (CAL) and bleeding on probing (BoP).

Periodontal diagnoses were assigned according to the current classification framework [12,27–29] and operationalized as described in Table 1.

**Table 1.** Diagnostic criteria for clinical periodontal conditions.

Condition	Periodontium status	Diagnostic criteria
Periodontal health	Intact	BoP < 10% of sites and no sites with PD > 3 mm; no clinical attachment loss or radiographic bone loss.
	Reduced	BoP < 10% of sites and no sites with PD > 3 mm; prior clinical attachment loss and/or bone loss present (e.g. successfully treated periodontitis), but currently stable.
Gingivitis	Intact	BoP ≥10% of sites and no sites with PD > 3 mm; no clinical attachment loss or bone loss.
	Reduced	BoP ≥10% of sites and no sites with PD > 3 mm; clinical attachment loss and/or bone loss present.
Periodontitis		clinical attachment loss associated with PD ≥4 mm at ≥2 non-adjacent interproximal sites; or clinical attachment loss ≥3 mm at ≥2 buccal/lingual (free) surfaces; with BoP.

### Microbial assessment

#### Collection of dental biofilm samples

Two biofilm samples were collected per participant in a fixed order, namely, supragingival first, then subgingival, using standardized techniques.

Supragingival plaque was collected from the buccal surface of the same tooth designated for subgingival sampling, using sterilized Gracey curettes (Hu-Friedy®, Chicago, IL, USA). The curette blade was positioned coronal to the gingival margin, and the plaque was removed with a single apico-coronal traction. If a participant did not meet the criteria for subgingival sampling (site with PD ≥4 mm, clinical attachment loss ≥3 mm, and bleeding on probing), only supragingival plaque was collected from a tooth with visible biofilm, preferentially a posterior tooth (molars to premolars, excluding third molars). If posterior teeth were absent, plaque was collected from an incisor; if no incisors were available, it was collected from a canine.

Subgingival plaque was collected from a single periodontal site with the greatest probing depth that met the selection criteria (PD ≥4 mm, clinical attachment loss ≥3 mm, bleeding on probing). Using sterilized Gracey curettes (Hu-Friedy®, Chicago, IL), the instrument was applied to the root surface and drawn apico-coronally to retrieve the subgingival sample.

Immediately after each collection, the active tip of the instrument was inserted into a pre-labelled microtube (participant ID and sample type: supragingival or subgingival) containing Tris-EDTA (TE) buffer, pH 8.0 (Ludwig Biotec®, Brazil). The tubes were transported to the laboratory and stored at −20 °C until analysis.

### DNA extraction and checkerboard DNA–DNA hybridization analysis

Genomic DNA was extracted using the Blood/Tissue DNA Mini Kit (Ludwig Biotec®, Brazil) following the manufacturer’s protocol. The levels and proportions of 40 bacterial species per sample were quantified using the checkerboard DNA–DNA hybridization method [30,31]. Briefly, aliquots were boiled for 10 min, then neutralized with 0.8 mL of 5 M ammonium acetate. The released DNA was loaded into the channels of a Minislot 30 apparatus (Immunetics, Cambridge, MA, USA) and immobilized onto a positively charged nylon membrane (15 × 15 cm; Boehringer Mannheim, Indianapolis, IN, USA).

Following prehybridization, species-specific DNA probes (for the 40 taxa) were generated using a random primer digoxigenin labelling kit (Boehringer Mannheim). Probes were applied perpendicular to the sample DNA lines using a Miniblotter 45, creating a checkerboard configuration (sample DNA horizontal; probes vertical). Hybridization was carried out at 42 °C for ≥20 h. Membranes were then removed, washed in a stringent wash solution, incubated with alkaline phosphatase-conjugated anti-digoxigenin antibody (Boehringer Mannheim), washed again, and developed in LumiPhos 530 substrate (Lumigen, Southfield, MI, USA) for 45  min at 37 °C.

For quantification, the chemiluminescent signal for each probe–sample intersection was compared to signals from two standards spotted on the same membrane corresponding to 10^5^ and 10^6^ bacterial cells. Signal intensities were converted to absolute counts by interpolation. Assay sensitivity was set to detect 10^4^ cells of a given species by adjusting the probe concentrations accordingly. These measurements were used to derive species levels and proportions within each supragingival and subgingival sample.

### Statistical analysis

Descriptive statistics were performed using absolute and relative frequencies for categorical variables and measures of central tendency for continuous variables. The Chi-squared test or Fisher’s exact test was applied to nominal variables to identify differences between groups according to clinical periodontal diagnosis (health, gingivitis and periodontitis) and cirrhosis severity (Child–Pugh and MELD scores). Associations between each oral condition variable and liver cirrhosis were estimated using odds ratios (OR) with 95% confidence intervals (CI). Analyses were conducted with SPSS version 21® (IBM Corp., Armonk, NY, USA), and statistical significance was set at *p* < 0.05.

For each bacterial species, the mean counts (×10⁵ cells) and the percentage of total DNA probe counts were calculated separately for supragingival and subgingival sites. These values were then averaged at the individual level and compared across clinical groups. The proportions of individual species were further aggregated to determine the relative distribution of microbial complexes.

Prior to group comparisons, outliers were identified and excluded within each group using the 1.5 × interquartile range (IQR) rule. Statistical analyses were performed using either the raw data or, when appropriate, data transformed by log10(x + 1) to stabilize variance.

For comparisons between two groups, Welch’s t-test was applied to account for potential heterogeneity of variances. For comparisons involving three or more groups, Welch’s one-way analysis of variance (Welch ANOVA) was used. When a significant main effect was detected, Games–Howell post hoc tests were performed to identify pairwise group differences.

For selected analyses, bacterial counts were log10(x + 1) transformed to stabilize variance and improve model performance. Because this transformation changes the scale of the data, the resulting values should be interpreted primarily as relative differences between groups rather than as direct representations of absolute bacterial counts.

To control for multiple testing across bacterial species, the Bonferroni correction was applied. All statistical analyses were conducted using Python (version 3.12.1), and the level of statistical significance was set at *p* < 0.05.

## Results

### Socio-demographic data

Patients were recruited, and samples were collected from July to December 2019. Of 246 patients with a diagnosis of liver cirrhosis under care at the clinic, 201 were excluded based on the study’s exclusion criteria. The final sample comprised 45 volunteers aged 23–71 years (mean 50.82, SD ± 12.61). Socio-demographic characteristics of the participants are summarized in Table 2.

**Table 2.** Sociodemographic characteristics of participants with liver cirrhosis (*n* = 45).

Variable	Category	n	%
Gender	Female	17	37.8
	Male	28	62.2
Skin colour	White	7	15.6
	Black	6	13.3
	Brown	31	68.9
	Prefer not to answer	1	2.2
Marital status	Single	14	31.1
	Married	23	51.1
	Stable union	2	4.4
	Divorced	5	11.1
	Widowed	1	2.2
Current employment status	Employed/active	8	17.8
	Not active	37	82.2
Education	Illiterate or functionally illiterate	10	22.2
	1–4 years	9	20
	5–8 years	17	37.8
	9–11 years	5	11.1
	≥12 years	4	8.9
Family income	Up to 1 minimum wage^*^	29	64.4
	>1 to ≤ 3 minimum wages	16	35.6

^*^ Minimum wage as defined by the national standard at the time of data collection (2019): R$ 998.00.

### Clinical data on liver condition

All participants had a diagnosis of liver cirrhosis, most commonly of nonalcoholic etiology (66.7%). Based on Child–Pugh classification, 71.1% were Class A, and 22.2% were Class B; in 6.7% of cases, the data were insufficient to assign a class. Regarding MELD, 77.8% had a score <15 and 17.8% had a score ≥15; two participants had insufficient data for MELD calculation. Additional clinical characteristics are summarized in Table 3.

**Table 3.** Clinical characteristics of participants with liver cirrhosis (*n* = 45).

Variable	Category	*n*	%
Etiology of cirrhosis	Alcoholic	15	33.3
	Nonalcoholic	30	66.7
Number of cirrhosis-related complications	0	18	40.0
	1	22	48.9
	2	3	6.7
	3	2	4.4
Comorbidities	Yes	30	66.7
	No	15	33.3
Current alcohol use	Yes	2	4.4
	No	43	95.6
Diuretic use	Yes	13	28.9
	No	32	71.1
Other medications	Yes	13	28.9
	No	32	71.1

Regarding the laboratory tests, the mean international normalized ratio (INR) of the sample was 1.27 (SD ± 0.21). The median platelet count was 85,500/µL (Q1: 47,750; Q3: 188,250). The median was reported due to the high variability in platelet counts (SD = 113,323.7).

### Oral behaviour and self-perception

Toothbrushing frequency ranged from rarely to twice daily in 53.3% of participants, and 57.8% reported no floss (interdental) use. Occasional or daily gingival bleeding was reported by 55.6%.

Dietary exposure to sugar-containing foods/beverages ≥3 times per day was reported by 75.6%, and 73.3% consumed soft drinks or artificial juice ≤ 2 times per week. In terms of dental attendance, 31.1% had a visit within the previous year, 26.7% within the previous 2 years, and 26.7% within the previous 3 years. Public dental services were used by 57.8%; 4.4% had never attended a dentist. The most frequent reason for the last dental visit was tooth extraction (35.6%), followed by routine examination/preventive care (26.7%) and other treatments (24.4%).

Tobacco exposure was reported by 51.1% (4.4% current; 46.7% former), whereas 48.9% were never-smokers. A perceived need for dental treatment was reported by 93.3%, and 68.9% expressed dissatisfaction with their oral condition. Most participants (82.2%) denied having a toothache in the preceding 6 months.

### Clinical findings on periodontal condition

Five participants (11.1%) met the criteria for periodontal health, 26 (57.8%) were classified as having gingivitis, and 14 (31.1%) were diagnosed with periodontitis, including 13 individuals with stage III grade B and one individual with stage IV grade B. All individuals classified as periodontally healthy or with gingivitis exhibited prior clinical attachment loss, which was consistent with a reduced periodontium. The quantitative periodontal clinical parameters are summarized in Table 4.

**Table 4.** Periodontal clinical parameters in participants with liver cirrhosis, by periodontal diagnosis (mean ± SD).

Variable	Health (*n* = 5)	Gingivitis (*n* = 26)	Periodontitis (*n* = 14)	Global
VPI (% of sites)	47.13 ± 24.59	65.72 ± 28.79	66.69 ± 30.52	63.95 ± 29.52
BoP (% of sites)	6.28 ± 3.39	36.52 ± 25.75	60.65 ± 28.27	40.67 ± 29.97
PD (mm)	1.44 ± 0.24	1.76 ± 0.28	2.21 ± 0.39	1.87 ± 0.40
CAL (mm)	1.88 ± 0.50	2.50 ± 0.79	3.04 ± 1.31	2.60 ± 1.03
GMP (mm)	0.45 ± 0.62	0.74 ± 0.80	0.85 ± 1.08	0.74 ± 0.89
# of sites PD ≤ 4mm (average)	0	0	4.07 ± 4.83	1.29 ± 3.28
# of sites PD ≤ 5-6 mm (average)	0	0	5.29 ± 4.95	1.64 ± 3.69
# of sites PD ≥7mm (average)	0	0	1.50 ± 2.10	0.47 ± 1.36

Abbreviations: VPI, visible plaque index; BoP, bleeding on probing; PD, probing depth; CAL, clinical attachment level; GMP, gingival margin position; SD, standard deviation.

Among participants with available liver-disease severity data, Child‒Pugh (*n* = 42) and MELD (*n* = 43), periodontal diagnoses differed by severity strata, as detailed in Table 5.

**Table 5.** Periodontal diagnosis by liver disease severity (Child–Pugh and MELD).

Periodontal diagnosis	Child–Pugh A *n* (%)	Child–Pugh B *n* (%)	Child–Pugh Total (*n*)	MELD < 15 *n* (%)	MELD ≥15 *n* (%)	MELD Total (*n*)
Health	4 (80.0)	1 (20.0)	5	5 (100.0)	0 (0.0)	5
Gingivitis	20 (83.3)	4 (16.7)	24	21 (87.5)	3 (12.5)	24
Periodontitis	8 (61.5)	5 (38.5)	13	10 (71.4)	4 (28.6)	14
Total	32 (76.2)	10 (23.8)	42	36 (83.7)	7 (12.3)	43

a. Child–Pugh: the classification could not be determined for 3 individuals because of insufficient clinical data. *p*-value of Fisher’s exact test = 0.681. OR gingivitis/health as a reference = 0.889 (95% CI 0.077–10.244). OR Periodontitis/Health as reference = 2.000 (95% CI 0.174–22.949). b. MELD: It was not possible to calculate the MELD severity score of the cirrhosis for 2 individuals. The *p*-value of Fisher’s exact test was 0.551. The OR was not calculated as there was a cell with a value of 0.

Periodontal diagnosis was not associated with liver-disease severity by either Child–Pugh (*p* = 0.681) or MELD (*p* = 0.551). Although patients classified as Child–Pugh B had higher odds of periodontitis versus Child–Pugh A (OR = 2.00; 95% CI, 0.174–22.949), this estimate was imprecise and not statistically significant. The BoP rate had a median of 39.1% (Q1 15.0%, Q3 60.8%), with no association with cirrhosis severity by Child–Pugh (*p* = 0.660) or MELD (*p* = 0.999).

### Microbiological findings

#### Supragingival biofilm

**Figure 1.**
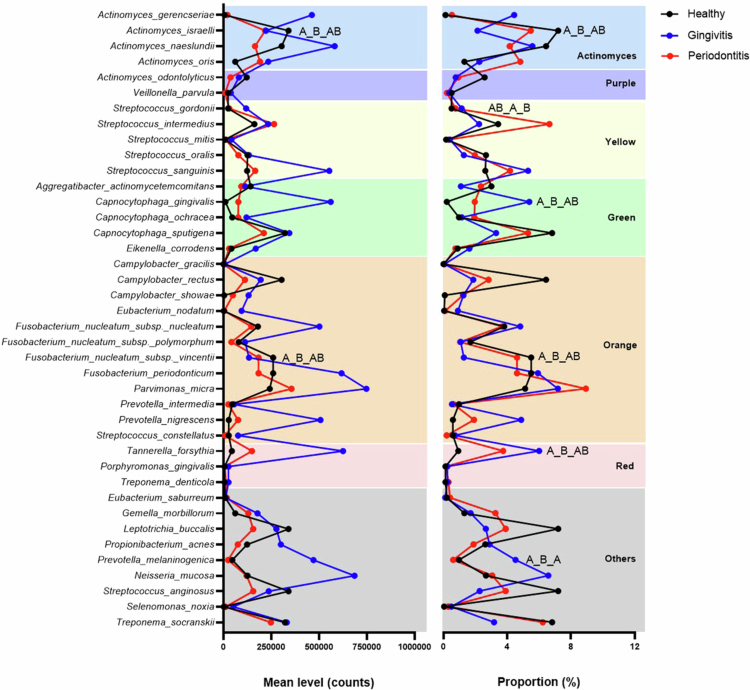
Counts of 40 bacterial species in supragingival biofilm from patients with periodontal health, gingivitis, and periodontitis. Letters indicate pairwise differences among groups (Welch’s ANOVA followed by Games–Howell post-hoc test, *p* < 0.05). For each species presenting statistical differences, three letters are shown in the order health–gingivitis–periodontitis; identical letters denote no difference, and different letters denote a significant difference.

In the supragingival biofilm, patients with gingivitis exhibited an overall shift toward increased bacterial burden and higher counts of disease-associated taxa when compared with periodontal health and periodontitis (Figure 1). This pattern was particularly evident for selected species within the orange and red complexes [30–32], which showed increasing trends from health to gingivitis, most prominently at the level of absolute counts. Among red complex organisms, *Tannerella forsythia* displayed the highest levels in the gingivitis group. Several orange complex species also demonstrated marked enrichment, especially in absolute counts in gingivitis; however, these differences did not reach statistical significance when compared with the other clinical groups. In contrast, health-associated taxa, particularly *Actinomyces israelii*, were more prominent in periodontally healthy individuals, both in counts and proportions, compared with the gingivitis group. Additional differences between groups were observed for early colonizers, including one species from the yellow complex, *Streptococcus gordonii*, which showed higher relative abundance in gingivitis compared with periodontitis, and a green complex species, *Capnocytophaga gingivalis*, which was more abundant in gingivitis than in health. *Fusobacterium nucleatum* ssp. *vincentii* exhibited higher counts and relative abundance in supragingival plaque from healthy individuals compared with gingivitis. *Prevotella melaninogenica* also varied across clinical conditions, showing higher proportional representation in gingivitis relative to both health and periodontitis. The relative abundance of the major Socransky’s complexes in supragingival plaque did not differ significantly among periodontal health, gingivitis, and periodontitis (Supplementary Figure 1).

**Figure 2.**
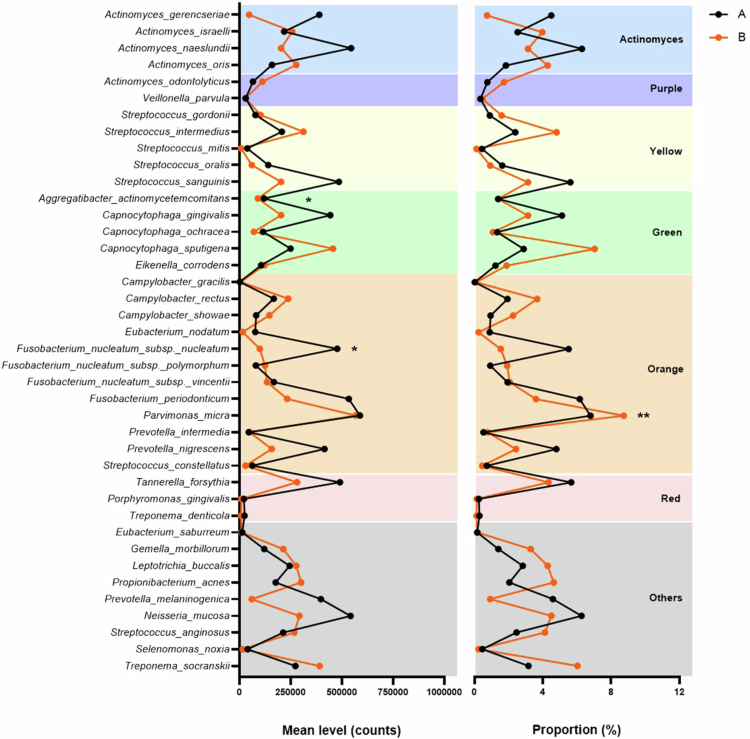
Counts (×10⁵) and relative proportions of the 40 microbial species in supragingival biofilm from Child–Pugh A and B patients. Asterisks (*) indicate species with significant differences between Child–Pugh classes [Welch’s t-test with Bonferroni correction, ** (*p* < 0.01), *(*p* < 0.05)].

When patients were distributed according to the Child–Pugh classification, the mean counts of *Aggregatibacter actinomycetemcomitans* (green complex) and *F. nucleatum* ssp. *nucleatum* (orange complex) were higher in Child–Pugh A than in Child–Pugh B (*p* < 0.05) (Figure 2). In terms of relative abundance, *Parvimonas micra* showed higher proportions in Child–Pugh B compared with Child–Pugh A (*p* < 0.01).

**Figure 3.**
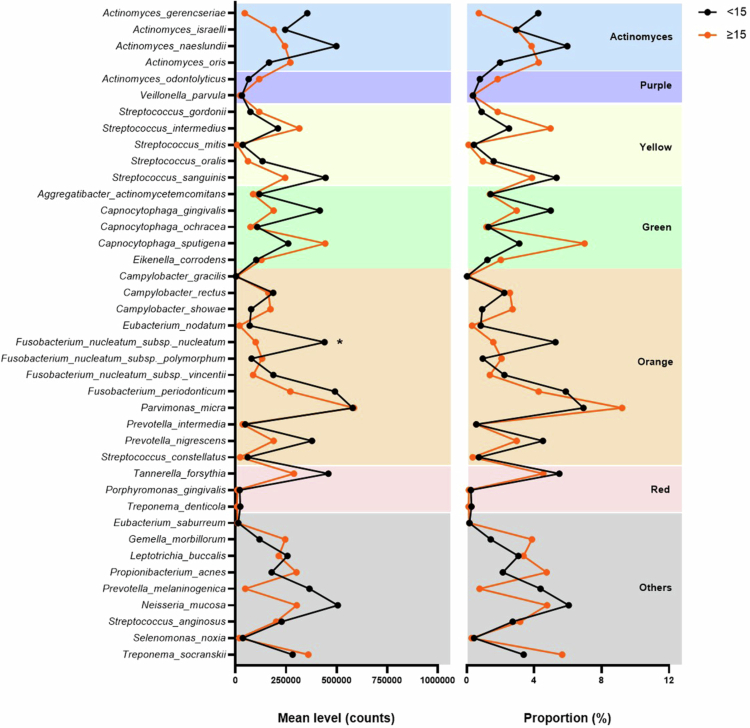
Counts (x10^5^) and relative proportions of the 40 microbial species in supragingival biofilm from patients with MELD < 15 and ≥15. Asterisks (*) indicate species with significant differences between MELD groups (Welch’s t-test with Bonferroni correction, *p* < 0.05).

When stratified by the MELD score, only the mean counts of *F. nucleatum.* ssp *nucleatum* (orange complex) were enriched in supragingival plaque from patients with MELD < 15 compared with those with MELD ≥15 (all *p* < 0.05) (Figure 3).

#### Subgingival biofilm

The proportions of bacterial complexes in the subgingival biofilm of patients with periodontitis are shown in Supplementary Figure 2. Overall, subgingival samples harboured similar counts and relative abundance across complexes in patients with different liver disease stages (Child–Pugh A and B – Supplementary Figure 3; MELD < 15 and MELD ≥15 – Supplementary Figure 4).

**Figure 4.**
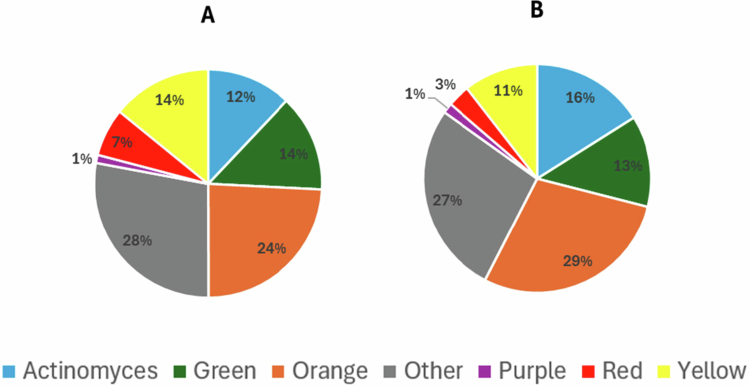
Distribution of Socransky’s complexes in subgingival plaque from periodontitis patients classified as Child–Pugh A and Child–Pugh B. Pie charts represent the relative abundance (%) of each complex. No statistically significant differences were observed between groups (Welch’s ANOVA followed by Games–Howell post-hoc test).

**Figure 5.**
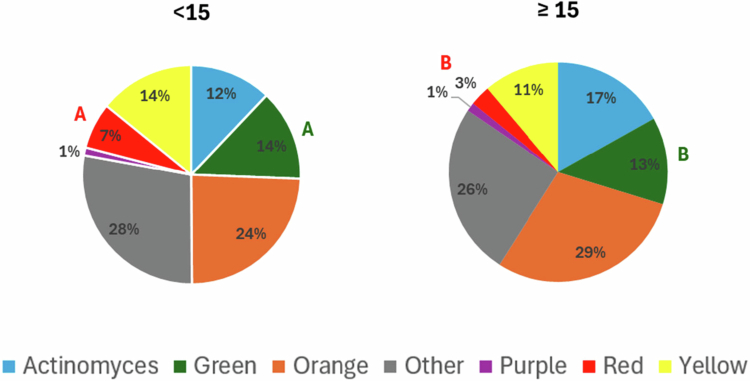
Distribution of Socransky’s complexes in subgingival plaque from periodontitis patients classified as MELD < 15 and ≥15. Pie charts represent the relative abundance (%) of each complex. Letters indicate significant differences by Welch’s ANOVA followed by Games–Howell post-hoc test (*p* < 0.05).

Across both cirrhosis severity stratification approaches (Child–Pugh classification and MELD score), patients with less advanced disease tended to exhibit lower proportions of red- and green-complex species in subgingival plaque; however, these differences reached statistical significance only when stratification was based on the MELD score (Figures 4 and 5).

## Discussion

A high burden of periodontal disease was observed in this population of cirrhotic patients, with 88.9% classified as having gingivitis or periodontitis; nonetheless, clinical periodontal diagnosis was not associated with liver-disease severity. Microbiological analyses revealed ecological shifts that did not follow a simple linear progression: in supragingival plaque, gingivitis was marked by the highest bacterial load and enrichment of orange- and red-complex pathogens. In subgingival plaque samples from patients with periodontitis, those with compensated liver disease (MELD < 15) harboured higher proportions of red- and green-complex pathogens than those with more advanced cirrhosis.

In cirrhosis, immune dysfunction, alterations in saliva composition, and haemostatic disturbances may reshape the oral microbial niches [33,34]. Within this context, the enrichment of orange- and red-complex species in supragingival plaque from gingivitis patients, particularly *Tannerella forsythia*, suggests that gingivitis represents a stage of increased supragingival dysbiosis. The lower representation of these taxa in periodontitis may reflect ecological reorganization as the disease progresses. The predominance of health-associated taxa, particularly *A. israelii*, in periodontally healthy individuals further supports a model of microbial succession from a commensal-dominated community toward dysbiosis [35].

An intriguing finding was that certain periodontal pathogens showed higher counts or proportions in patients with less severe liver disease. In supragingival plaque, for example, *A. actinomycetemcomitans* and *F. nucleatum* ssp. *nucleatum* were enriched in patients with compensated cirrhosis, while in subgingival plaque from periodontitis patients, those with MELD < 15 exhibited higher proportions of red complex pathogens than those with MELD ≥15. Several non-mutually exclusive explanations may explain this pattern. Patients with more advanced cirrhosis may have received more intensive medical management, including systemic antibiotics or other interventions that alter the periodontal microbiota. In addition, cirrhosis-associated immunoparesis may alter host–microbe interactions, such that periodontal destruction can occur in response to less pathogenic microbial communities that would otherwise be tolerated in immunocompetent hosts [36,37]. This interpretation is supported by Jensen et al. [20], who also reported reduced detection of classical periodontal pathogens in the subgingival microbiota of patients with periodontitis and cirrhosis.

Additional species-level findings merit comment. *S. gordonii,* a yellow-complex early colonizer, showed higher relative abundance in gingivitis compared with periodontitis, consistent with its role in initial biofilm formation [38]. *C. gingivalis* was more abundant in gingivitis than in health, potentially reflecting transitional community dynamics [39]. Similarly, *P. melaninogenica* was proportionally enriched in the gingivitis relative to the other two groups, suggesting a potential role in gingivitis-associated dysbiosis. While not formally recognized as a keystone pathogen, its proteolytic and community-modulating properties may influence early community shifts [40]. Interestingly, *F. nucleatum* ssp. *vincentii* exhibited higher counts and relative abundance in supragingival plaque from healthy individuals compared with gingivitis, suggesting that not all *Fusobacterium* subspecies follow identical ecological trajectories [41]. In addition, the supragingival enrichment of orange- and red-complex species observed in gingivitis is consistent with the reservoir concept proposed by Ximénez-Fyvie et al. [32] and Feres et al. [42], suggesting that supragingival plaque may serve as a reservoir of pathogenic potential that precedes or parallels subgingival colonization.

Despite the well-established concept that oral dysbiosis not only contributes to local periodontal tissue destruction but also may serve as a source of systemic inflammatory burden [43], including hepatic disease, major gastroenterology and hepatology guidelines rarely address oral health in preventive or therapeutic recommendations. The findings of the present study provide clinical and microbiological support for the frequent coexistence of periodontal disease and liver cirrhosis and help strengthen the link between these fields, offering a framework for future investigations. The high prevalence of gingivitis and periodontitis observed in this population (88.9%), together with the microbiological patterns, supports the integration of periodontal surveillance into hepatology care. In patients with compensated liver disease, the supragingival enrichment of pathogenic taxa during gingivitis suggests that this stage may represent a clinically relevant window for targeted preventive strategies, warranting evaluation in future interventional studies. In patients with decompensated cirrhosis or those approaching transplantation, pre-procedural optimization and bleeding-aware dental protocols remain essential, even though bleeding on probing was not associated with liver-disease severity.

Several limitations should be acknowledged. The relatively small sample size and the limited number of individuals in some severity strata require that subgroup analyses be interpreted cautiously. In particular, the small periodontally healthy subgroup may have limited statistical power for some comparisons. In addition, our outpatient, single-centre study population was enriched for patients with compensated cirrhosis, which may limit its generalizability to more decompensated populations. The cross-sectional design of this study precludes causal inference regarding whether liver dysfunction drives microbial shifts or whether periodontal dysbiosis contributes to hepatic disease progression. Therefore, the relationships observed between periodontal status, microbial composition, and liver disease severity should be interpreted as associative rather than directional.

Despite these limitations, this study has several important strengths. To our knowledge, this is the first study to simultaneously evaluate both supragingival and subgingival microbial niches in a population with liver cirrhosis, with analyses stratified by two standard measures of liver-disease severity, the Child–Pugh classification and the MELD score. In addition, we quantified a panel of 40 bacterial species using checkerboard DNA–DNA hybridization, a technique with more than two decades of successful application in periodontal research [30,31]. These species have been consolidated and validated across numerous clinical studies as representative microbial signatures of periodontal health and disease, supporting the biological relevance and interpretability of the observed microbial patterns [44,45].

## Conclusion

In this population with cirrhosis, periodontal disease was highly prevalent, with gingivitis on a reduced periodontium representing the most common clinical presentation. Although periodontal diagnosis was not associated with liver-disease severity, distinct microbiological patterns emerged. Gingivitis was characterized by increased supragingival dysbiosis, whereas more advanced liver disease was not associated with a higher pathogen burden – possibly reflecting the impact of medical management or altered host‒microbe interactions in cirrhosis. These findings suggest that host factors associated with liver disease may influence periodontal microbial ecology.

Together, these findings generate hypotheses regarding the interplay between periodontal microbial ecology and liver-disease severity, with the gingivitis stage warranting particular attention in future research. Longitudinal and interventional studies, specifically designed to clarify the nature, directionality, and clinical implications of the periodontal-liver axis, are needed to confirm the ecological dynamics observed.

## Supplementary Material

Supplementary Material Batista et al 2026_SUPPLEMENTARY MATERIAL FINAL.docx

## Data Availability

The data supporting the findings of this study are not publicly available due to ethical and privacy restrictions involving human participants.
