# Supplementary material for: Periodontal status and microbial profiles across the clinical spectrum of liver cirrhosis
Source: J Oral Microbiol. 2026 May 26;18(1):2678639. doi: 10.1080/20002297.2026.2678639 (PMC13218307; doi:10.1080/20002297.2026.2678639)
Supplement: Supplementary Material — Batista et al 2026_SUPPLEMENTARY MATERIAL FINAL.docx [file ZJOM_A_2678639_SM6313.docx]

**SUPPLEMENTARY MATERIAL**

**
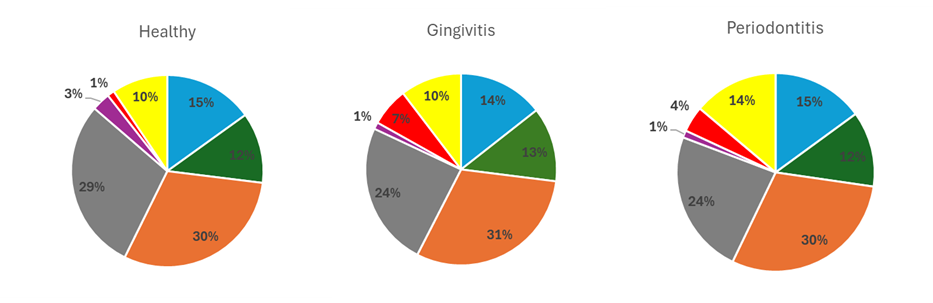
**

**
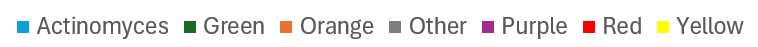
**

**Supplementary Figure 1.** Distribution of Socransky’s complexes in supragingival plaque according to periodontal diagnosis (periodontal health, gingivitis, and periodontitis). Pie charts represent the relative abundance percentages of each complex within each clinical group. No statistically significant differences in proportional distribution were observed among groups (Welch’s ANOVA followed by Games–Howell post hoc test, p>0.05).


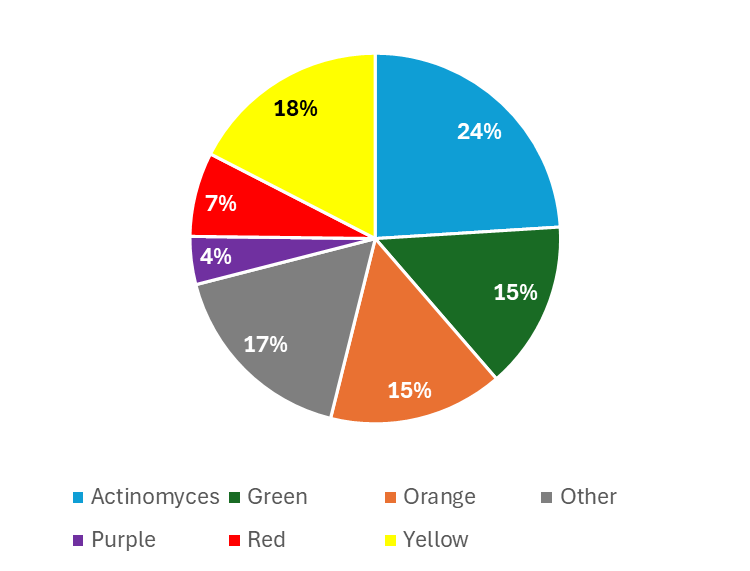


**Supplementary Figure 2.** Distribution of Socransky’s complexes in subgingival plaque from patients with periodontitis. The pie chart represents the relative abundance (%) of each complex.


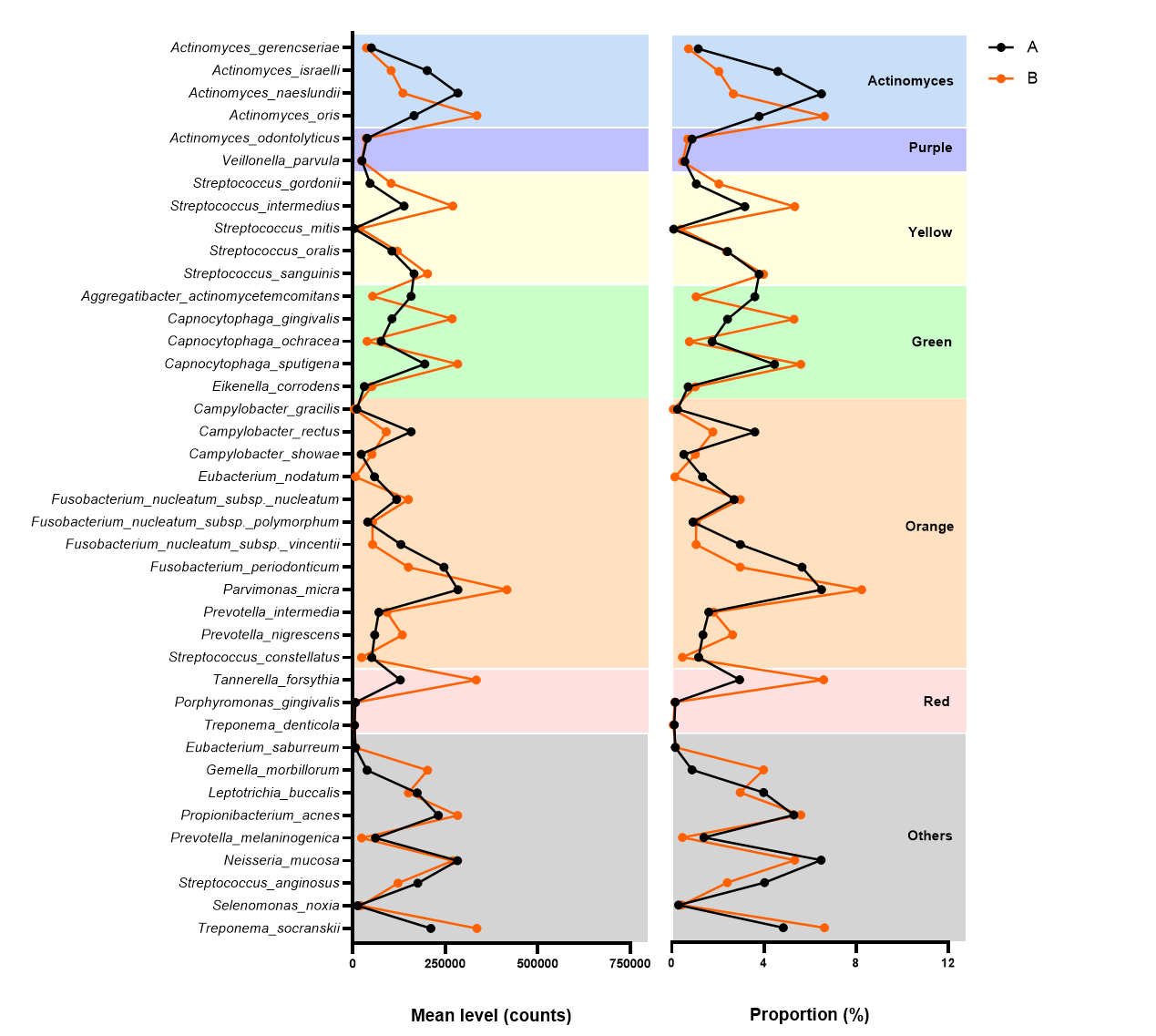


**Supplementary Figure 3.** Microbiological composition of subgingival biofilm by species in Child–Pugh A and B patients: counts and relative proportions. No statically significant differences were observed between the groups (Welch’s t-test with Bonferroni correction, p>0.05).


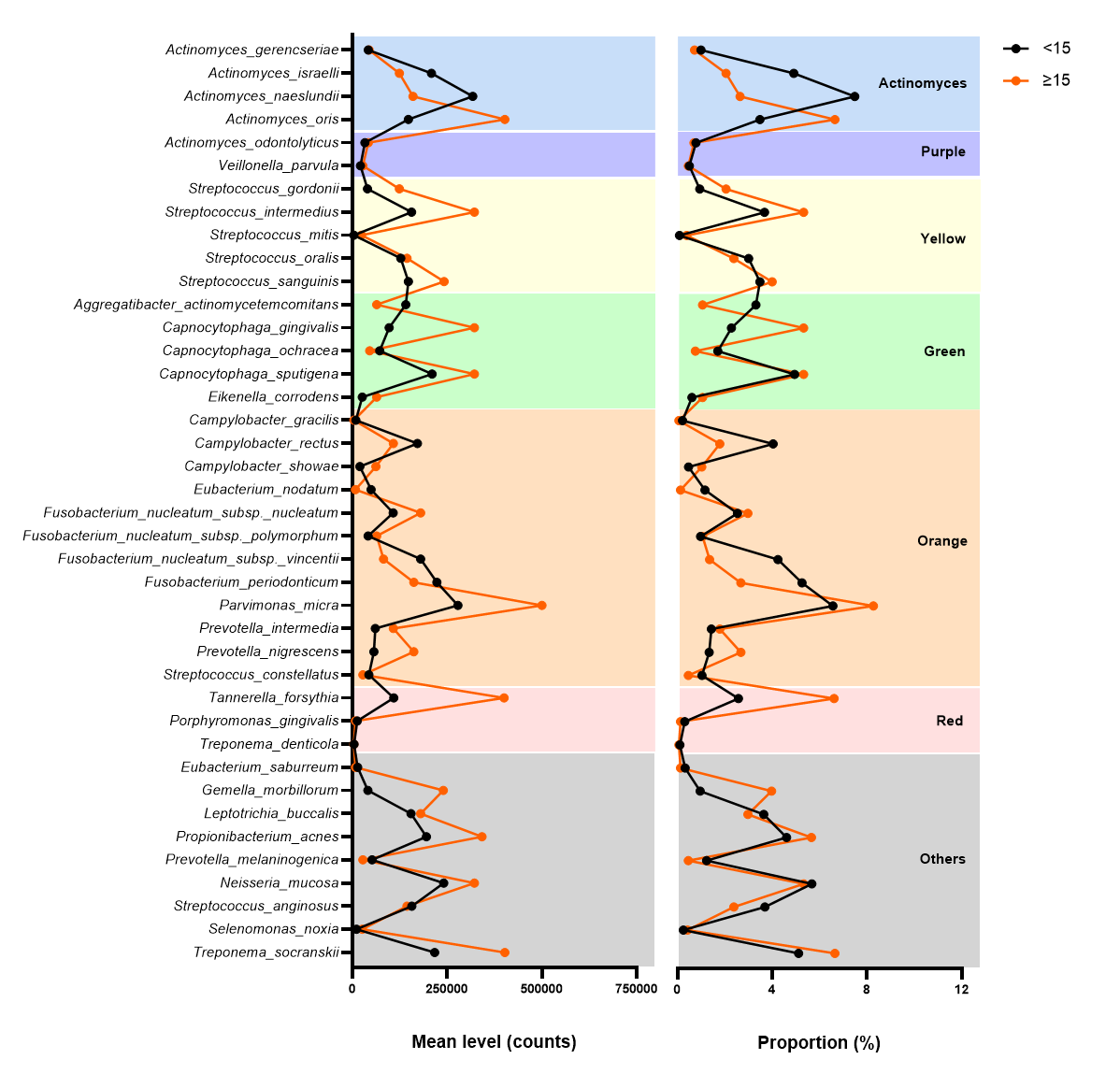


**Supplementary Figure 4.** Microbiological composition of subgingival biofilm by species in patients with MELD <15 and ≥15: counts and relative proportions. No statically significant differences were observed between the groups (Welch’s t-test with Bonferroni correction, p>0.05).
